# NSCLC: Current Evidence on Its Pathogenesis, Integrated Treatment, and Future Perspectives

**DOI:** 10.3390/jcm14031025

**Published:** 2025-02-06

**Authors:** Kareem Tahayneh, Mayar Idkedek, Firas Abu Akar

**Affiliations:** 1 Faculty of Medicine, Al-Quds University, East Jerusalem 20002, Palestine; ktahayna@gmail.com; 2 Department of General Surgery, Faculty of Medicine, Al-Quds University, East Jerusalem 20002, Palestine; 3 Department of Thoracic Surgery, The Edith Wolfson Medical Center, Holon 58100, Israel; 4 Sackler Faculty of Medicine, Tel Aviv University, Tel Aviv 6997801, Israel

**Keywords:** lung cancer, NSCLC, pathogenesis, screening, combination therapy, personalized treatment

## Abstract

Non-small cell lung carcinoma (NSCLC) comprises the majority of lung cancer cases, characterized by a complex interplay of genetic alterations, environmental factors, and molecular pathways contributing to its pathogenesis. This article highlights the multifaceted pathogenesis of NSCLC and discusses screening and integrated strategies for current treatment options. NSCLC is an evolving field with various aspects including the underlying molecular alterations, oncogenic driver mutations, and immune microenvironment interactions that influence tumor progression and response to therapy. Surgical treatment remains the most applicable curative option, especially in the early stages of the disease, adjuvant chemotherapy may add benefits to previously resected patients. Combined Radio-chemotherapy can also be used for palliative purposes. There are various future perspectives and advancing horizons in NSCLC management, encompassing novel therapeutic modalities and their applications, such as CAR-T cell therapy, antibody-drug conjugates, and gene therapies. On the other hand, it’s crucial to highlight the efficacy of innovative modalities of Immunotherapy and immune checkpoint inhibitors that are nowadays widely used in treatment of NSCLC. Moreover, the latest advancements in molecular profiling techniques and the development of targeted therapies designed for specific molecular alterations in NSCLC play a significant role in its treatment. In conclusion, personalized approaches are a cornerstone of successful treatment, and they are based on a patient’s unique molecular profile, tumor characteristics, and host factors. Entitling the concept of individualized treatment strategies requires proper patient selection, taking into consideration mechanisms of resistance, and investigating potential combination therapies, to achieve the optimal impact on long-term survival.

## 1. Introduction

Lung cancer is considered the leading cause of mortality in the United States. According to the American Cancer Society’s report, the estimated newly diagnosed cases and deaths of lung cancer in 2024 are expected to be 234,580 and 125,070, respectively in the United States [1].

Eighty percent of lung cancer cases are classified as NSCLC and only 20% are considered SCLC. Furthermore, the incidence and mortality rates of lung cancer have been decreasing throughout the past years, which may be attributed to reductions in smoking, earlier detection of the disease, and the recent advances in NSCLC treatment options. As for the 5-year relative survival rate for lung cancer, it is estimated as 25% overall, higher in women (30%) than men (21%), and it is also higher in NSCLC (30%) than the SCLC (8%) [1].

Risk factors of lung cancer can be classified into modifiable, such as smoking, secondhand smoke, exposure to radon or asbestos or other carcinogens, and non-modifiable, including exposure to previous radiation therapy to the lungs, personal or family history of lung cancer, and air pollution [1].

Throughout the years, multiple studies have been investigating the role of screening in reducing mortality in lung cancer patients. Clinical trials showed no reduction in lung cancer mortality when screening through chest radiographs with or without cytologic analysis of sputum specimens [2]. 

Afterward, efforts have been shifted to using low-dose helical computed tomography (CT) as a lung cancer screening modality for high-risk individuals. The U.S. Preventive Services Task Force recommended the annual screening using low-dose CT for lung cancer in all asymptomatic individuals aged 55–80 years old who have a smoking history of 30 pack-years and currently smoke or former smokers who had quit smoking in the previous 15 years [3].

The National Lung Screening Trial (NLST) aimed at identifying the role of low-dose CT in reducing lung cancer-associated mortality. 53,454 participants at high risk for lung cancer were enrolled in the low-dose CT and chest radiograph groups. Results have shown that there were an overall 24.2% and 6.9% positive results in low-dose CT and radiographs, respectively. Although there were high rates of false-positive results in both groups, especially in the low-dose CT group, the incidence of lung cancer was higher in the low-dose CT group. Moreover, there was a 20% reduction in the lung cancer mortality rate in the low-dose CT group [4].

Furthermore, Europe’s largest, Dutch-Belgian Lung Cancer Screening trial (NELSON) aimed to investigate if low-dose CT screening reduced lung cancer mortality by more than 26% [5]. Results showed that lung cancer deaths were significantly lower among participants who had undergone screening with CT. Similarly, the MILD trial has shown a 39% and 20% reduction in lung cancer mortality at 10 years and overall mortality rates, respectively, when compared to the control group [6,7].

On the other hand, there were several challenges to the use of CT scans in lung cancer screening, including overdiagnosis, high false-positive rates, cost-effectiveness, long-term radiation risks, and stress due to prolonged follow-up [8].

## 2. Literature Review

### 2.1. Non-Small Cell Lung Cancer: Definition & Pathophysiology

Non-small cell lung cancer (NSCLC) is defined as any type of epithelial lung cancer other than small cell lung cancer (SCLC). It most commonly constitutes squamous cell carcinoma, large cell carcinoma, and adenocarcinoma [9]. 

Adenocarcinoma is the most common type of NSCLC and usually arises from type II alveolar cells in the lungs’ periphery [10]. Squamous cell carcinoma is the second most common type and arises from squamous cells in the airway epithelial cells in the center of the lungs. Large cell carcinoma is usually a diagnosis of exclusion, where there’s no existence of squamous or glandular maturation [11].

Epithelial type II alveolar cells are often involved in lung tissue regeneration, and as aforementioned they’re often implicated in NSCLC progression because of their susceptibility to mutations such as the KRAS and EGFR [12]. Additionally, all cancer stages; including metastasis, are related to fibroblasts. Cancer-associated fibroblasts secrete extracellular matrix and growth factors, which enhance tumor invasion and progression [13]. Moreover, different cell types enhance cancer progression by suppressing immune response and enhancing tissue invasion, these include: regulatory t-cells, tumor-associated neutrophils, and tumor-associated macrophages [13,14,15].

The pathophysiology of NSCLC involves several key processes; initiation and promotion, in which carcinogens lead to alteration in cells and genetic mutations. Afterward, these genetic mutations may result in the activation of oncogenes or the inactivation of tumor suppressor genes that ultimately lead to uncontrolled cell growth and tumor development. The most commonly identified mutations are Epidermal Growth Factor Receptor (EGFR), Anaplastic Lymphoma Kinase (ALK), and Kirsten Rat Sarcoma Virus (KRAS). Moreover, dysregulation of the cellular signaling pathways leads to further progression of the disease, and lastly, NSCLC tumors induce angiogenesis, metastasis, and immune evasion [16,17,18,19,20].

Likewise, NSCLC is believed to be the result of genetic, epigenetic, and environmental factors. The pathogenesis of NSCLC is thought to be a progressive process, in which smoking for example plays a vital role as a lung carcinogen. It is believed that some types of NSCLC have premalignant lesions that undergo morphological changes before progressing into cancer, including hyperplasia, metaplasia, dysplasia, and carcinoma in situ [9].

In 2004, the first mutations were identified in the epidermal growth factor receptor (EGFR) and additive specific gain in function that later explained the clinical responsiveness to gefitinib [21]. Since then, there have been substantial efforts in identifying new molecular alterations in lung cancer patients aiming at developing targeted and personalized therapy.

Identifying these molecular alterations will not only help in providing targeted therapies, but also in the disease’s prognosis. A retrospective study held on 1095 patients in Korea concluded that EGFR is an independent risk factor for recurrence, and patients who had EGFR mutations had higher rates of distant metastasis when compared to wild-type EGFR [22].

### 2.2. Non-Small Cell Lung Cancer: Clinical Presentation & Diagnosis

The Twenty-Year Retrospective of Lung Cancer (TYROL study) that included 2293 consecutive NSCLC patients aimed to describe the NSCLC patients’ characteristics. Unfortunately, 37.9% of the patients were diagnosed with stage IV [23].

NSCLC usually presents with a variety of symptoms; most commonly including cough (54.7%), dyspnea (45.3%), pain, weight loss, pneumonia, night sweats, hemoptysis, or even can be diagnosed incidentally in asymptomatic patients [23]. 

Furthermore, the clinical manifestations of lung cancer may arise from local invasion, compression to adjacent structures, or distant metastasis, such as hoarseness when compressing the vocal cords, and personality changes in cases of brain metastases [9].

Diagnosis of NSCLC is a multi-step process that constitutes accurate history taking and physical examination, laboratory evaluation, Chest X-ray, chest CT scan or PET-CT scan, and finally obtaining biopsies [9,24].

Biopsies are often needed for the diagnosis of NSCLC and histologic confirmation, in addition to the need to determine the extent of the disease to further define the TNM stage, which eventually directs the treatment options [25].

Several treatment options are available for lung cancer. However, some factors must be taken into consideration to determine the best treatment option, which include; tumor size, location, and margins. The involvement of surrounding tissue is also crucial; pleural invasion and lymph node invasion focus on the number and location of involved LN, as well as the lymphovascular invasion. All of these factors are used to determine the stage of the tumor, which guides decision-making alongside histological type and molecular features [9].

### 2.3. Overview of Currently Available Treatment Options

Surgery remains the cornerstone in the NSCLC management, however, a recent clinical trial on IB to III A tumors showed that neoadjuvant nivolumab plus chemotherapy resulted in longer event-free survival than chemotherapy alone [26]. There are different available surgical options, depending on the size of the existing tumor and the local spread of the mass, which further require a personalized and tailored approach for each patient. The surgical options range from sub-lobar resection that includes wedge or segmental resections and could be as wide as lobar resection [27]. Furthermore, NSCLC could be also managed by Lobectomy and mediastinal lymphadenectomy. Extensive involvement of the majority of the lung or difficulties in preserving lobes limits the previous preservative choices and may require Pneumonectomy, however, Pneumonectomy should be avoided whenever it is possible since it was confirmed that bronchial and vascular sleeve resections have better outcomes when compared with pneumonectomy [9,28].

To maximize the surgical resection’s outcomes, some studies recommended post-surgery adjuvant chemotherapy to eliminate any remaining cancer cells and reduce the chance of recurrence. In addition, the ADAURA clinical trial has revealed that the use of adjuvant Osimertinib (a third-generation epidermal growth factor receptor tyrosine kinase inhibitor (EGFR-TKI)) was associated with prolongation of disease-free benefit, reduction of the risk of local and distant recurrence, and efficiency in EGFR-mutated NSCLC, as compared to placebo [29,30].

On the other hand, for patients with tumors that cannot be surgically resected, radiation therapy is considered a valuable treatment option. When combined with chemotherapy, it can offer long-term survival benefits and is also used to alleviate symptoms such as pain and breathing difficulties. Additionally, stereotactic radiotherapy (SBRT) was found to have a significantly higher five-year survival rate than conventional radiotherapy [31].

As for advanced or metastatic NSCLC, chemotherapy can enhance survival rates and quality of life. Platinum-based drugs, often used in combination with other medications, are common chemotherapeutic agents used in the management of NSCLC [32,33].

The role of targeted therapy has been studied in various articles due to its ability to predict the response to treatment. Advances in understanding genetic mutations in NSCLC have led to the development of targeted therapies. Medications that target EGFR mutations, ALK rearrangements, and other specific genetic changes offer significant benefits, including extended survival and better symptom control [9,17].

Moreover, immunotherapy has efficiently transformed the NSCLC treatment. Immune checkpoint inhibitors, like PD-1/PD-L1 inhibitors, have shown great potential in treating advanced disease stages by boosting the body’s immune response against cancer cells [34]. 

Synergistic effects of combination therapy have been confirmed to result in improved patient outcomes compared to either modality alone. The practice of combining various treatment modalities, such as chemotherapy with targeted therapy or immunotherapy, is becoming more common nowadays due to its promising outcomes [35].

### 2.4. Surgical Management: VATS vs. RATS

Video-Assisted Thoracoscopic Surgery (VATS) and Robotic-Assisted Thoracoscopic Surgery (RATS) have revolutionized the surgical management of non-small cell lung cancer (NSCLC), offering minimally invasive alternatives to traditional open thoracic surgery.

VATS has become a standard approach for the surgical resection of early-stage NSCLC due to its minimally invasive nature. Only with small incisions and a thoracoscope, lobectomies and other lung resection procedures can be successfully performed. Despite special requirements of training and experience of the surgeon, studies have shown that VATS provides several advantages over traditional open surgery, including reduced postoperative pain, shorter hospital stays, quicker recovery times, and fewer complications compared to open thoracotomy. Moreover, VATS has been associated with non-inferior oncological outcomes to open surgery, making it a preferred choice for many thoracic surgeons [36,37].

RATS utilizes robotic systems to enhance precision and control during surgery. This approach allows for better articulation and visualization which make it superior to conventional VATS in terms of challenging anatomical situations. RATS has shown promise in improving the accuracy of tumor resection and lymph node dissection, which are critical for effective cancer treatment. Despite the higher costs and longer operative times associated with RATS, it has been linked to better postoperative outcomes and fewer complications in some studies [38,39,40].

The future of VATS and RATS in NSCLC treatment seems promising with ongoing advancements in technology and techniques. Research is focusing on further minimizing invasiveness and improving surgical precision for improved patient outcomes. Developments such as reducing the number of incisions to one in single port VATS enhanced robotic supportive technologies including imaging and better instrumentation, and uniportal RATS are likely to advance the frontiers of minimally invasive surgery [41]. Additionally, the recruiting of artificial intelligence and machine learning tools in surgical planning and execution could improve these techniques further [42,43].

Both VATS and RATS represent significant strides in the surgical management of NSCLC, providing patients with effective, less invasive options that promote quicker recovery and better quality of life. As technology continues to evolve, these methods are likely to become even more refined and widely adopted, further improving the outcomes for patients with NSCLC. Surgery can also be utilized for difficult biopsies that are not accessible via CT-guided or trans-bronchial approach, or for palliative treatment as Talcage.

### 2.5. Treatment Guidelines for NSCLC Stages & Follow-Up

Locoregional staging of the tumor can be determined based on the revised 8th tumor, node, and metastasis (TNM) classification of malignant tumors that was published by the Union for International Cancer Control (UICC) [44]. Clinical staging and pretreatment pathological diagnosis in stages I-III are crucial steps before establishing the treatment plan for NSCLC. The European Society for Medical Oncology (ESMO) published the following clinical practice guidelines in 2017, and updates in 2021 [45,46].

For Stage I disease, lobectomy has been the standard treatment for resectable tumors. However, for patients unable to tolerate surgery, stereotactic body radiotherapy (SBRT) is an option. Adjuvant Therapy is typically not required unless high-risk features are present. 

As for Stage II NSCLC, lobectomy or pneumonectomy are the available options similar to Stage I NSCLC, in addition to chemotherapy; typically cisplatin-based; which is often recommended post-surgery to reduce the recurrence risk.

Stage III and locally advanced tumors require a multimodal approach that combines surgery, chemotherapy, and radiotherapy. Chemo-radiotherapy is often recommended for unresectable tumors, and concurrent chemo-radiotherapy is the standard. Durvalumab may be given as consolidation therapy post-chemo-radiation [47].

Moreover, the management of Stage IV tumors includes systemic therapy; Targeted therapy (e.g., EGFR inhibitors, ALK inhibitors), or immunotherapy (e.g., PD-1/PD-L1 inhibitors) depending on the molecular profiling. Additionally, palliative care is crucial to manage the patient’s symptoms and improve their quality of life. Radiation therapy may also be used to alleviate symptoms.

It is worth mentioning that all patients who receive a radical intent may develop new-cancer-related problems and should be followed up by a multidisciplinary team for treatment-related complications, recurrence of lung cancer, or detection of other primary tumors. For the first 2 years post-treatment, a follow-up visit every 6 months is recommended. The follow-up should include a thorough history, physical examination, and a contrast-enhanced volume chest and abdominal CT scan at least at 12 and 24 months, with an optional PET-CT scan if required. Afterwards, an annual visit is often recommended aiming at the detection of second primary tumors [45,46].

Furthermore, palliative care is an indispensable and essential part of the management of NSCLC patients. Various Palliative support options are available for metastatic and advanced NSCLC, early and integrated application of palliative care is not meant primarily to improve survival, however, it has a crucial role in reducing patients’ physical and psychological burdens. This results in an improved quality of life, a better understanding of prognosis, and less aggressive care that usually takes place at the end of life. Providing Palliative care concurrent with standard cancer-directed care is feasible and acceptable with better overall results including physical, mood, and results showing longer survival in patients who received early palliative care as compared with those receiving standard care alone [48,49].

The most commonly treated cancer-related problems in palliative care are fluid buildup in regional spaces and airway obstruction. Pleural effusion can be managed by either multiple drainage through thoracocentesis or by permanent catheter placement, while chemical or surgical pleurodesis serves as a method that prevents recurrence. Pericardial effusion is also managed either by pericardiocentesis or by surgery creating a pericardial window. Another implementation of palliative care in NSCLC is in treating dyspnea and pneumonia that are caused by tumors blocking the airways, sometimes it’s treated with radiotherapy but recently with photodynamic therapy or Laser therapy, especially in the early stages of lung cancer. In advanced cases and larger tumors that cause more significant obstruction, stent placement is used to obtain patency of airways, it’s either used alone or along with the previously mentioned methods [9].

### 2.6. Novel Therapeutic Options for NSCLC Management

The future of NSCLC treatment is promising, with significant advancements in several areas:

#### 2.6.1. Advancements in Surgical Techniques

Minimally invasive surgical techniques, such as VATS and (RATS), continue to evolve. These approaches offer reduced postoperative pain, shorter hospital stays, and faster recovery times compared to traditional open surgery. The future holds potential improvements in robotic technology and the incorporation of artificial intelligence to enhance surgical precision and outcomes.

#### 2.6.2. Molecular and Genetic Profiling; Targeted Therapies

Recent advances in the treatment of NSCLC have centered on targeted therapies and their integration with other treatment modalities to enhance efficacy and overcome resistance. The integration of comprehensive molecular and genetic profiling in clinical practice is set to revolutionize personalized therapy for NSCLC. By identifying specific genetic mutations and alterations, such as EGFR, ALK, and ROS1, targeted therapies can be more precisely tailored to individual patients. This personalized approach aims to enhance treatment efficacy and minimize adverse effects.

Single-agent therapies provide approaches based on specific molecular alterations. For instance, BRAF inhibitors like vemurafenib and dabrafenib have demonstrated efficacy in patients with BRAF V600E mutations. These inhibitors can be administered alone, especially when combination therapy is not viable due to patient-specific factors. The introduction of targeted therapies like crizotinib, an ALK inhibitor, has transformed NSCLC management. Crizotinib has shown remarkable efficacy in NSCLC patients harboring ALK rearrangements. Crizotinib binds to the ALK kinase domain, inhibiting tumor cell proliferation and survival. It is also effective against ROS1 rearrangements, offering another therapeutic avenue [50].

Similarly, osimertinib, a third-generation EGFR inhibitor, is a standard therapy for EGFR-mutated NSCLC. Its ability to target the T790M resistance mutation distinguishes it from earlier EGFR inhibitors. These single-agent therapies not only extend progression-free survival but also improve the quality of life for patients with specific genetic mutations [51].

#### 2.6.3. Immunotherapy

Immunotherapy has emerged as a cornerstone in the management of advanced NSCLC. Agents like pembrolizumab and nivolumab, which target PD-1/PD-L1 pathways, have shown substantial benefits in improving overall survival. These agents restore the immune system’s ability to recognize and target cancer cells. Pembrolizumab, in particular, has demonstrated superior efficacy as a first-line treatment for advanced NSCLC expressing PD-L1, highlighting the importance of biomarker-driven treatment [52]. Ongoing research focuses on discovering new immune checkpoint inhibitors and developing combination therapies to overcome resistance and improve outcomes.

#### 2.6.4. Combination Therapies

The combination of different treatment modalities, including chemotherapy, targeted therapy, and immunotherapy, is being actively explored. Studies are investigating the synergistic effects of combining these treatments to improve efficacy and overcome resistance. This multimodal approach aims to provide a more comprehensive attack on cancer cells while minimizing the risk of recurrence.

While single-agent therapies have shown promise, combining targeted agents with other treatment modalities has emerged as a strategy to overcome resistance and improve efficacy. A notable example is the combination of EGFR inhibitors with VEGF inhibitors, such as bevacizumab. This approach targets both tumor cell proliferation and angiogenesis, providing dual inhibition that has shown enhanced efficacy in clinical trials [53]. VEGF inhibitors, such as bevacizumab, disrupt tumor angiogenesis, while EGFR inhibitors like osimertinib or erlotinib target tumor cell proliferation. This combination has shown improved outcomes by interfering with the formation of new blood vessels and cellular signaling pathways essential for tumor growth [54].

Another promising strategy is the use of immune checkpoint inhibitors, such as pembrolizumab or nivolumab, in combination with chemotherapy. Chemotherapy induces immunogenic cell death, which amplifies the immune system’s response when combined with checkpoint inhibitors. This approach has been particularly beneficial for NSCLC patients, regardless of their PD-L1 expression status, thus broadening the scope of immunotherapy’s potential [55].

The combination of ALK inhibitors, like lorlatinib, with inhibitors of other pathways, such as CDK4/6 inhibitors, also holds promise for delaying resistance and enhancing tumor control. By targeting multiple survival pathways, this approach provides a more comprehensive attack on tumor biology [56].

Similarly, the addition of MET pathway inhibitors, such as tepotinib, to EGFR inhibitors has shown potential in overcoming resistance driven by MET amplification. This combination addresses both the primary and resistance-associated mechanisms in EGFR-mutant NSCLC, further broadening therapeutic possibilities [57].

Additionally, the integration of radiotherapy with immune checkpoint inhibitors has been explored for its synergistic effects. Radiotherapy enhances antigen presentation and immune activation, complementing the immune-enhancing properties of checkpoint blockade and offering a novel therapeutic synergy [58].

### 2.7. Limitations of the Current NSCLC Treatment Options

According to the most recent global cancer report, lung cancer is still the leading cause of mortality among 36 other cancer types, and it’s the second most frequently diagnosed cancer type worldwide [59]. The rising rates of lung cancer mortality and morbidity have led to increased efforts to understand its pathophysiology.

Genetic mutations and increasing genetic instability are preliminary to developing cancer, which leads to heterogeneous manifestations of NSCLC, hence, a more complex composition of NSCLC, and a more difficult treatment [60,61].

Drug resistance poses a significant challenge in the treatment of NSCLC, often undermining the long-term effectiveness of therapies. Resistance can be classified as either primary, where the tumor fails to respond initially, or acquired, which develops after an initial phase of treatment success. A prominent mechanism of resistance is genetic alterations within cancer cells. For example, patients treated with EGFR inhibitors, such as osimertinib, may develop secondary mutations like the T790M mutation, reducing the drug’s ability to bind and inhibit the target protein. Similarly, resistance to ALK inhibitors like crizotinib often arises due to mutations in the ALK gene or activation of bypass signaling pathways that sustain tumor growth despite the therapy [50,51,57]. Phenotypic changes in tumors also contribute to resistance. A common example is the histological transformation of NSCLC into small-cell lung cancer, a subtype less amenable to targeted therapies [62,63].

Moreover, although surgical resection is considered a preferred treatment in early-stage NSCLC, a retrospective study held in Japan showed a 19.7% recurrence rate after complete surgical resection, with EGFR mutations being an independent risk factor for recurrence [22]. Another review article described a 30–55% recurrence rate of NSCLC after curative resection. Recurrence rates can be attributed to incomplete resection microscopically and macroscopically, underestimation of the true tumor stage due to micro-metastatic cancer cells that are usually not detected using the used staging methods, and dissemination of cancer cells during surgical handling [64]. Moreover, a more than 12-week delay from radiographic diagnosis to surgery was associated with increased recurrence rates and worse survival in stage I NSCLC [65].

Using chemotherapy as a neoadjuvant treatment or in metastatic lung cancer is challenging due to many factors including; treatment failure and increasing resistance most probably due to genetic and epigenetic alterations of tumor cells, in addition to its systemic toxicity sometimes hinders its use, including myelosuppression, neuropathy, gastrointestinal disturbances and others [62,66].

Radiation therapy is considered a cornerstone in the treatment of NSCLC especially in combined therapy or in patients who aren’t surgical candidates, however, its use is sometimes limited due to the increased risk of complications that limit its effectiveness. Tumor hypoxia is one of the factors that decrease the sensitivity to radiotherapy due to decreased reactive oxygen species [67]. Other complications include its serious side effects, such as cardiotoxicity, arrhythmias, and heart failure which occur in 20% of patients with lung cancer [68]. In one cohort study published in 2023, a substantial increase in the risk of second primary solid malignancies and gastrointestinal cancer was observed in lung cancer patients who received radiotherapy [69].

On the other hand, recent studies have described the development of progressive drug resistance to targeted therapies, which leads to reduced efficacy. Resistance mechanisms were classified as on-target resistance which is due to mutations in target structure, or off-target resistance which includes activation of alternative pathways [70]. Targeted therapies, such as EGFR and ALK inhibitors, often cause skin toxicities, including acneiform rash, as well as diarrhea and fatigue. Rare but severe complications, such as cardiac toxicity with ALK inhibitors, can limit their use. Immune checkpoint inhibitors, like pembrolizumab, are associated with immune-related adverse events resulting from an overactive immune response. These effects can manifest as dermatological issues (rash, pruritus), gastrointestinal problems (colitis and diarrhea), endocrine dysfunctions (thyroiditis, adrenal insufficiency), and respiratory complications such as pneumonitis, which can be life-threatening [71].

## 3. Discussion

This review article highlights the NSCLC pathogenesis and the complex interplay of genetic, environmental, and molecular factors. Several articles confirm the role of genetic mutations, such as EGFR, ALK, and KRAS, in the progression of NSCLC [20]. The significant roles of NSCLC screening and early detection in addition to surgical resection, and emerging targeted therapies in its management are also emphasized in this article. Additionally, this review highlights the promising role of personalized medicine in optimizing treatment outcomes based on individual molecular profiles.

The integration of findings from various studies provides a comprehensive understanding of NSCLC’s pathogenesis and treatment. Previous studies have shown that genetic mutations, such as those in EGFR and ALK, play a crucial role in NSCLC development and progression [16,17,18,19,20,22]. These findings have led to the development of targeted therapies that improve survival rates and symptom control. Moreover, the evolution of surgical techniques, VATS, and RATS, has significantly improved patient outcomes and quality of life by reducing postoperative pain, and hospital stays, and enhancing recovery times [36,37,39,40].

Advances in screening methods, such as low-dose computed tomography (LDCT), have improved the early detection of NSCLC. Using LDCT has significantly decreased mortality rates in the NLST. Research into biomarkers and liquid biopsies also holds promise for less invasive and more accurate screening methods in the future [4,6,7].

Various NSCLC treatment approaches have been studied throughout the literature, while surgical resection remains the cornerstone of resectable tumors [9], one study has recommended the use of adjuvant chemotherapy to maximize the surgery’s effects [30]. Otherwise, radiation therapy, alone or in combination with chemotherapy, is usually used in patients whose surgery can’t fully resect the tumor. It offers evidenced long-term survival benefits [31].

Additionally, targeted therapy and immunotherapy have a promising role in offering improved patient outcomes. The combination of different treatment modalities, including chemotherapy, targeted therapy, and immunotherapy, holds the potential to provide a more comprehensive and effective approach to NSCLC management [35].

Despite the advancements in pathogenesis understanding and treating NSCLC, several limitations remain. For instance, while targeted therapies offer significant benefits, they are often limited to patients with specific genetic mutations, leaving a substantial proportion of patients without these mutations reliant on traditional chemotherapy and radiotherapy [9,17]. 

Therefore, Chimeric antigen receptor (CAR) T-cell therapy is emerging as a promising strategy in treating various cancers, including NSCLC. CAR-T therapy works by modifying a patient’s T cells to express a chimeric antigen receptor (CAR) that targets specific cancer cells. Upon re-administration, these engineered T cells recognize and bind to tumor-associated antigens, triggering an immune response to kill the tumor cells. Although traditionally more successful in hematologic cancers, CAR T-cell therapy is now being tested in solid tumors, where challenges like the tumor microenvironment and antigen heterogeneity persist. In the context of NSCLC, the primary challenge is enhancing CAR T cells’ ability to overcome the tumor’s obstacles and successfully target solid tumor antigens, potentially establishing CAR T therapy as a groundbreaking treatment. Recent studies highlight its potential in NSCLC treatment, focusing on modifying CAR T cells to overcome barriers such as improving the targeting of tumor-specific antigens and boosting T-cell persistence. As research progresses, CAR T therapy may provide a transformative treatment option for NSCLC, complementing or enhancing existing therapies [72,73].

The LIFE study examines the economic burden of non-small cell lung cancer (NSCLC) patients; this study highlights the significant direct and indirect costs associated with NSCLC, including medical expenses, loss of productivity, and financial strain on patients and their families. It finds that NSCLC significantly strains both the healthcare system and patients’ financial resources. The study suggests that improved treatment strategies and cost-effective management could mitigate some of the financial pressures faced by patients, underlining the need for efficient healthcare policies [74].

While significant progress in the management of NSCLC has been made, research gaps remain in the literature that warrant further investigation. The long-term effectiveness and potential resistance mechanisms associated with new targeted therapies and immunotherapies need to be thoroughly examined. Furthermore, there is a need for more studies focusing on the cost-effectiveness and broader accessibility of advanced surgical techniques and personalized treatment strategies since the associated higher costs may limit their accessibility, particularly in developing countries.

Future research should aim to address the identified gaps by exploring the long-term outcomes of new therapies. Studies should also investigate the integration of cost-effective strategies to make advanced treatments more accessible.

## 4. Conclusions

In conclusion, this review article highlights the NSCLC pathogenesis and the evolving landscape of its treatment. Moreover, it discusses the importance of screening and early detection of lung cancer using low-dose CT scans despite its possible risks. The currently available treatment options for NSCLC are various, and they depend on the clinical stage of the tumor, it also discusses the novel therapeutic options, combination therapies, and their limitations. While new drugs and treatment combinations are continually being developed and tested in clinical trials, including next-generation targeted therapies and novel immunotherapeutic agents, ongoing cancer research and management plans are now being focused on personalized treatment approaches. These approaches are based on the genetic profile of the patient, aiming at optimizing the therapy’s effectiveness and minimizing its side effects. This is because personalized approaches are considered one of the most promising treatment options that are crucial for optimizing treatment outcomes. Continuous research and clinical trials are crucial to translating these advancements into real benefits for patients, ultimately aiming to improve survival rates and quality of life.

## Data Availability

No new data were created or analyzed in this study.
